# Comparison of Perioperative Single-Dose Systemic Dexamethasone Versus Placebo for Prolongation of Postoperative Analgesia in Term Parturients Undergoing Cesarean Section Under Spinal Anesthesia

**DOI:** 10.7759/cureus.90011

**Published:** 2025-08-13

**Authors:** Nivedha Ravishankar, Sriraam Kalingarayar, Suriyaraj Govarthanan

**Affiliations:** 1 Anesthesiology, Kovai Medical Center and Hospital, Coimbatore, IND; 2 Anesthesiology, West Suffolk NHS Foundation Trust, Bury St Edmunds, GBR; 3 Anesthesiology, PSG Institute of Medical Sciences and Research, Coimbatore, IND

**Keywords:** analgesics, anesthesia, anti-inflammatory agents, cesarean section, dexamethasone, pain, postoperative, spinal

## Abstract

Background and objective

Postoperative pain management following cesarean section under spinal anesthesia remains a significant clinical challenge, with limited analgesic duration frequently necessitating rescue interventions. Dexamethasone, a synthetic corticosteroid with established anti-inflammatory and analgesic properties, has demonstrated potential for prolonging neuraxial anesthesia effects. This randomized controlled trial (RCT) aimed to evaluate the efficacy of perioperative single-dose systemic dexamethasone versus placebo for prolongation of postoperative analgesia in term parturients undergoing cesarean section under spinal anesthesia, with secondary assessment of antiemetic and anti-shivering effects.

Methodology

This RCT was conducted at a tertiary care hospital over 12 months, recruiting 60 American Society of Anesthesiologists physical status grade II (ASA PS II) parturients aged 20-35 years undergoing elective cesarean section under spinal anesthesia. Participants were randomly allocated using the envelope method into two groups of 30 patients each. The dexamethasone group received intravenous dexamethasone 0.1 mg/kg diluted to 2 mL, while the placebo group received 2 mL normal saline as a placebo, administered 15 minutes before spinal anesthesia. Standardized spinal anesthesia was performed using 2 mL hyperbaric bupivacaine 0.5% with 20 mcg fentanyl. Postoperative assessment included visual analog scale (VAS) pain scores, sensory and motor blockade characteristics, and incidence of nausea, vomiting, and shivering, monitored every 30 minutes for 24 hours.

Results

Demographic characteristics demonstrated no statistically significant differences between groups, with mean ages of 25.70 ± 3.45 years in the dexamethasone group versus 25.90 ± 2.82 years in the placebo group. Dexamethasone administration significantly prolonged postoperative analgesia, with statistically significant VAS score differences observed from 240 to 750 minutes postoperatively. At 360 minutes, mean VAS scores were 0.00 ± 0.00 in the dexamethasone group versus 3.47 ± 1.46 in the placebo group (13.3%), representing peak analgesic difference. Sensory blockade regression demonstrated significant prolongation at 90 minutes and 210 minutes in the dexamethasone group. Motor blockade recovery showed no significant inter-group differences, with complete recovery achieved by 270 minutes in both groups. Postoperative nausea and vomiting incidence were 13.3% (n = 4) in the dexamethasone group versus 20% (n = 6) in the placebo group, although not statistically significant. Shivering occurred in one (3.3%) patient in the dexamethasone group versus none in the placebo group.

Conclusion

Perioperative single-dose intravenous dexamethasone 0.1 mg/kg significantly prolongs postoperative analgesia following cesarean section under spinal anesthesia, extending analgesic duration by approximately 270 minutes with an excellent safety profile. These findings support routine clinical implementation for enhanced maternal comfort and reduced rescue analgesic requirements.

## Introduction

Postoperative pain management following cesarean section remains a critical challenge in obstetric anesthesia, significantly impacting maternal recovery, early mobilization, and quality of life. The administration of neuraxial techniques, particularly spinal anesthesia with intrathecal local anesthetics and adjuvants, provides excellent intraoperative conditions but frequently results in limited postoperative analgesic duration, typically ranging from 2 to 4 hours with conventional bupivacaine dosing, necessitating early rescue analgesic interventions. Contemporary literature demonstrates that inadequate postoperative pain control following cesarean delivery can lead to prolonged hospital stays, delayed maternal-infant bonding, and increased risk of chronic pain development [1,2].

Dexamethasone, a synthetic corticosteroid with potent anti-inflammatory and analgesic properties, has emerged as a promising perioperative intervention for enhancing postoperative analgesia across various surgical procedures. Recent systematic reviews and meta-analyses have established the efficacy of both perineural and systemic dexamethasone administration in prolonging analgesic duration and reducing opioid requirements [3-5]. Specifically in obstetric populations, multiple randomized controlled trials (RCTs) have demonstrated that intravenous dexamethasone administration significantly extends the duration of spinal anesthesia-induced analgesia, with analgesic benefits persisting for 8-12 hours postoperatively [6-8].

The mechanistic basis for dexamethasone's analgesic efficacy involves multiple pathways, including suppression of inflammatory mediator release, blockade of nociceptive C-fiber transmission, and enhanced local anesthetic potency through membrane stabilization effects [9]. Contemporary clinical investigations have demonstrated that single-dose intravenous dexamethasone (0.1-0.2 mg/kg) administered perioperatively significantly reduces postoperative pain scores, extends time to first analgesic request, and decreases total rescue analgesic consumption following cesarean section under spinal anesthesia [10-12].

Furthermore, dexamethasone demonstrates favorable secondary effects, including reduction in postoperative nausea and vomiting incidence, attenuation of postoperative shivering, and enhancement of overall patient satisfaction scores [13,14]. Recent meta-analytic evidence consistently supports the safety profile of single-dose perioperative dexamethasone administration in obstetric populations, with minimal adverse effects and no significant impact on neonatal outcomes [15,16].

Despite compelling evidence supporting dexamethasone's analgesic efficacy, standardized protocols for optimal dosing, timing, and patient selection criteria remain inadequately defined in obstetric anesthesia practice. This RCT was designed to evaluate the efficacy of perioperative single-dose systemic dexamethasone versus placebo for prolongation of postoperative analgesia in term parturients undergoing cesarean section under spinal anesthesia, with secondary assessment of antiemetic and anti-shivering effects.

## Materials and methods

Study design and study setting

This investigation employed an RCT design to evaluate the efficacy of perioperative single-dose systemic dexamethasone for prolongation of postoperative analgesia in term parturients undergoing cesarean section under spinal anesthesia. The study was conducted at Kovai Medical Center and Hospital, Coimbatore, India, a 750-bedded multispecialty tertiary care facility with National Accreditation Board for Hospitals and Healthcare Providers (NABH) accreditation.

Study period

The clinical investigation was conducted over a 12-month period from September 2019 to September 2020, allowing adequate patient recruitment and comprehensive data collection across diverse seasonal variations and clinical scenarios.

Ethics committee approval

Prior to participant recruitment, comprehensive ethical approval was obtained from the hospital ethics committee in accordance with the Declaration of Helsinki principles and Good Clinical Practice guidelines. Written informed consent was secured from all participants following a detailed explanation of study procedures, potential risks, benefits, and voluntary participation principles. Participants retained the right to withdraw from the study at any time without compromising their clinical care.

Sample size estimation

Sample size determination employed systematic power analysis incorporating empirical parameters from Shahraki et al. (2013), who conducted a double-blind randomized clinical trial examining intravenous dexamethasone effects in 60 parturients undergoing cesarean section [17]. The calculation utilized the standard formula:

$n = \frac{z^2_{1-\alpha/2}(2\sigma^2_p)}{d^2}$

where σ²p represents pooled variance derived from visual analog scale (VAS) pain score distributions across temporal measurement intervals. Shahraki et al. demonstrated VAS score standard deviations ranging from 0.8 to 2.9 in treatment groups and from 2.2 to 4.0 in control groups, with clinically meaningful differences of 1.3-3.4 points. Comprehensive temporal variance analysis across early (0-120 minutes), intermediate (150-420 minutes), and extended (450-1440 minutes) postoperative periods yielded maximum required sample sizes of 13 participants per group. Statistical parameters included type I error α = 0.05, type II error β = 0.20 (80% power), and 95% confidence intervals (CIs). To ensure robust statistical validity and accommodate potential attrition, the final sample size was established at 30 participants per group (total number: 60).

Inclusion criteria

Eligible participants included term parturients aged 20-35 years classified as American Society of Anesthesiologists physical status grade II (ASA PS II), scheduled for elective lower segment cesarean section under spinal anesthesia. All participants demonstrated normal obstetric parameters with singleton pregnancies at term gestation, ensuring homogeneous study population characteristics and minimizing confounding variables.

Exclusion criteria

Excluded participants included those with febrile conditions, fetal distress requiring emergency intervention, contraindications to spinal anesthesia necessitating general anesthesia, obesity with a body mass index (BMI) exceeding 35 kg/m^2^, present or previous history of peptic ulcer disease, glaucoma, diabetes mellitus, heart failure, failure of spinal anesthesia requiring conversion to general anesthesia, and current intravenous steroid therapy. While not exhaustive, these primary exclusion criteria addressed major confounding variables affecting analgesic outcomes while maintaining clinical feasibility. Additionally, participants were excluded post-randomization if they demonstrated inadequate sensory blockade (below T6 level at 10 minutes), required intraoperative supplemental analgesia or sedation, experienced conversion to general anesthesia, or developed intraoperative complications requiring deviation from standard protocol.

Sampling method

Participants were recruited using systematic random sampling methodology with envelope-based randomization to ensure unbiased group allocation. During the 12-month study period, 72 eligible parturients were screened for participation. Of these, eight did not meet inclusion criteria (five with BMI > 35 kg/m^2^, two with contraindications to spinal anesthesia, and one with pre-existing diabetes mellitus), and four declined participation, citing preference for alternative analgesic protocols. The remaining 60 participants were randomly assigned to one of two groups using the sealed envelope technique, maintaining allocation concealment and preventing selection bias. Double-blinding procedures were implemented with both participants and outcome assessors remaining unaware of group assignments throughout the study period.

Intervention

The dexamethasone group participants received intravenous dexamethasone 0.1 mg/kg diluted to 2 mL total volume, while the placebo group participants received 2 mL normal saline as a placebo control. Interventions were administered by preoperative nursing staff approximately 15-20 minutes before anticipated spinal anesthesia, acknowledging variability in actual timing based on clinical workflow. Standardized spinal anesthesia was performed under strict aseptic conditions with participants positioned in the right lateral decubitus position. The medical team accessed the subarachnoid space using a 26-gauge Quincke-Babcock needle after identifying the presumed L3-L4 interspace through palpation of anatomical landmarks and local infiltration with 2 mL of 2% lignocaine. The intrathecal injection consisted of 2 mL hyperbaric bupivacaine 0.5% (10 mg) combined with 20 mcg fentanyl, administered slowly with confirmation of cerebrospinal fluid flow. To address potential confounding from anthropometric variations, spinal anesthetic dosing was standardized at 2 mL hyperbaric bupivacaine 0.5% (10 mg) with 20 mcg fentanyl for all participants, acknowledging this location as a study limitation that may influence individual analgesic duration based on height and weight differences. This represents our institutional standard protocol for cesarean delivery, providing reliable surgical anesthesia with an established safety profile.

Data collection procedure

Postoperative monitoring was conducted systematically for 24 hours following surgical completion. Visual analog scale (VAS) pain assessments were performed every 30 minutes during hours 0-12 and then hourly during hours 13-24, using the standardized 0-10 numeric rating scale [18]. Two trained anesthesia technicians alternated eight-hour shifts (07:00-15:00, 15:00-23:00, and 23:00-07:00), conducting assessments, with nursing staff providing supplementary monitoring during technician breaks. The pain assessment protocol specified evaluation at rest, with participants instructed to rate pain intensity without movement provocation. Sensory blockade regression was assessed by cold sensation at dermatomal levels T4-T12 bilaterally at 30-minute intervals until complete resolution. Motor blockade recovery was assessed using the modified Bromage scale at identical intervals [19,20].

Postoperative nausea and vomiting (PONV) were documented as present/absent (binary classification), with ondansetron 4 mg administered intravenously for persistent symptoms (>15 minutes duration or patient request). Shivering was assessed using the Bedside Shivering Assessment Scale, with meperidine 12.5 mg administered for grade ≥2 shivering. However, participants requiring anti-shivering medication (n = 1) were excluded from primary analysis to prevent confounding, as meperidine possesses analgesic properties. Rescue analgesia protocol specified diclofenac 75 mg intramuscularly for VAS ≥ 3 or patient request, with eight-hour minimum intervals between doses. All assessment personnel remained blinded to group allocations throughout the observation period.

Data analysis

Statistical analysis was performed using the Statistical Package for the Social Sciences (SPSS) version 26 software package (IBM Corp., Armonk, NY). Descriptive statistics included mean and standard deviation (SD) calculations for continuous variables, while categorical variables were expressed as frequencies and percentages. Inferential statistical testing employed an independent samples t-test for continuous variable comparisons between groups, while a chi-square test was utilized for categorical variable associations. Statistical significance threshold was established at p < 0.05 for all comparative analyses.

## Results

Table 1 shows the demographic and clinical parameters of the study cohort, demonstrating comparable baseline characteristics between treatment groups. The dexamethasone group had a mean age of 25.70 ± 3.45 years (range: 20-34 years), while the placebo group had 25.90 ± 2.82 years (range: 21-35 years), with no statistically significant difference (p = 0.807). Anthropometric measurements revealed mean heights of 155.03 ± 4.61 cm in the dexamethasone group versus 154.87 ± 5.75 cm in the placebo group (p = 0.902). Weight distribution showed 69.87 ± 7.08 kg in the dexamethasone cohort compared to 65.85 ± 8.42 kg in the placebo group (p = 0.050). Body mass index calculations demonstrated 29.12 ± 3.16 kg/m^2^ in the dexamethasone group versus 27.46 ± 3.29 kg/m^2^ in the placebo group (p = 0.052). While weight and BMI demonstrated borderline statistical significance, the mean differences of 4.017 kg in weight and 1.650 kg/m^2^ in BMI represent clinically negligible variations unlikely to influence spinal anesthetic distribution or analgesic outcomes. Gravidity averaged 2.1 ± 0.8 in the dexamethasone group versus 2.2 ± 0.9 in the placebo group (p = 0.651), indicating similar previous pregnancy exposure. Parity demonstrated 0.9 ± 0.7 in the dexamethasone cohort compared to 1.0 ± 0.8 in the placebo cohort (p = 0.602), reflecting equivalent prior delivery experiences. Gestational age at delivery showed 38.5 ± 1.1 weeks in the dexamethasone group versus 38.7 ± 0.9 weeks in the placebo group (p = 0.441), confirming all participants underwent term cesarean deliveries. Previous cesarean section history was present in eight (26.7%) participants in the dexamethasone group and nine (30%) participants in the placebo group (p = 0.500), indicating balanced distribution of repeat cesarean deliveries. These findings establish homogeneous baseline characteristics between study groups, minimizing confounding variables and enhancing internal validity for subsequent comparative analyses.

**Table 1:** Baseline Demographic and Clinical Characteristics Data presented as mean ± standard deviation unless otherwise specified Continuous variables analyzed using an independent samples t-test; categorical variables analyzed using a chi-square test Statistical significance threshold: p < 0.05 BMI: body mass index, SD: standard deviation

Parameter	Dexamethasone group (n = 30)	Placebo group (n = 30)	Mean difference	Statistical test	p-value
Age (years)					
Mean ± SD	25.70 ± 3.45	25.90 ± 2.82	0.200	t = -0.246	0.807
Range	20-34	21-35			
Anthropometric parameters					
Height (cm)	155.03 ± 4.61	154.87 ± 5.75	0.167	t = 0.124	0.902
Weight (kg)	69.87 ± 7.08	65.85 ± 8.42	4.017	t = 2.000	0.050
BMI (kg/m^2^)	29.12 ± 3.16	27.46 ± 3.29	1.650	t = 1.980	0.052
Obstetric parameters					
Gravidity	2.1 ± 0.8	2.2 ± 0.9	0.100	t = -0.454	0.651
Parity	0.9 ± 0.7	1.0 ± 0.8	0.100	t = -0.514	0.602
Gestational age (weeks)	38.5 ± 1.1	38.7 ± 0.9	0.200	t = -0.772	0.441
Previous cesarean (number (%))	8 (26.7)	9 (30)	-	Χ^2^ = 0.082	0.500

The comprehensive temporal analysis in Table 2 demonstrates the analgesic efficacy profile of perioperative dexamethasone administration across the 24-hour postoperative observation period. Both groups exhibited identical pain-free intervals from 0 to 180 minutes postoperatively. Significant divergence emerged at 240 minutes, with the dexamethasone group maintaining pain-free status while the placebo group demonstrating emerging pain (0.27 ± 0.69, p = 0.039). Peak analgesic divergence occurred at 420 minutes, with the dexamethasone group remaining pain-free versus the placebo group experiencing maximum pain intensity (3.83 ± 0.91, p < 0.001). Notably, the dexamethasone group exhibited delayed pain onset, first reporting discomfort at 510 minutes (0.40 ± 0.81), while maintaining significantly lower pain scores throughout the observation period. An intriguing biphasic pattern emerged in the dexamethasone group, with pain scores peaking at 660 minutes (4.00 ± 0.98) before declining, contrasting with the placebo group's earlier peak and subsequent reduction. Statistical significance persisted through 750 minutes and re-emerged at late measurement points (1,260 and 1,290 minutes), demonstrating sustained analgesic benefits.

**Table 2:** VAS Pain Scores: Postoperative Period Data presented as mean ± standard deviation ^*^Statistical significance at p < 0.05 Significant analgesic effect observed from 240 to 750 minutes postoperatively VAS: visual analog scale

Time point (minutes)	Dexamethasone group	Placebo group	Mean difference	t-value	p-value
0-180	0.00 ± 0.00	0.00 ± 0.00	-	-	-
210	0.00 ± 0.00	0.13 ± 0.51	0.133	-1.439	0.155
240	0.00 ± 0.00	0.27 ± 0.69	0.267	-2.112	0.039^*^
270	0.00 ± 0.00	0.93 ± 1.46	0.933	-3.500	<0.001^*^
300	0.00 ± 0.00	1.80 ± 1.79	1.800	-5.511	<0.001^*^
330	0.00 ± 0.00	2.47 ± 1.53	2.467	-8.858	<0.001^*^
360	0.00 ± 0.00	3.47 ± 1.46	3.467	-13.042	<0.001^*^
390	0.00 ± 0.00	3.77 ± 1.19	3.767	-17.274	<0.001^*^
420	0.00 ± 0.00	3.83 ± 0.91	3.833	-23.000	<0.001^*^
450	0.00 ± 0.00	3.47 ± 0.94	3.467	-20.262	<0.001^*^
480	0.00 ± 0.00	3.10 ± 0.89	3.100	-19.191	<0.001^*^
510	0.40 ± 0.81	2.77 ± 0.82	2.367	-11.241	<0.001^*^
540	1.13 ± 1.43	2.47 ± 0.82	1.333	-4.427	<0.001^*^
570	1.43 ± 1.28	2.27 ± 0.87	0.833	-2.954	0.005^*^
600	3.53 ± 1.25	1.93 ± 0.69	1.600	6.127	<0.001^*^
660	4.00 ± 0.98	1.87 ± 0.73	2.133	9.544	<0.001^*^
720	3.13 ± 1.14	1.83 ± 0.75	1.300	5.236	<0.001^*^
750	2.53 ± 0.78	1.77 ± 0.73	0.767	3.946	<0.001^*^
1,260	1.93 ± 0.37	1.67 ± 0.61	0.267	2.063	0.044^*^
1,290	1.91 ± 0.32	1.70 ± 0.54	0.267	2.343	0.023^*^

The analysis in Table 3 examines the temporal progression of sensory blockade regression, revealing differential recovery patterns between treatment groups. At 90 minutes postoperatively, the dexamethasone group demonstrated superior sensory preservation, with five (16.7%) participants maintaining T4 level blockade compared to 0 (0%) participants in the placebo group. The majority of dexamethasone participants exhibited T5 (10 (33.3%) participants) and T6 (15 (50%) participants) sensory levels, while placebo group participants predominantly demonstrated T6 regression (26 (86.7%) participants), indicating more rapid sensory recovery (p = 0.002). At 150 minutes, the dexamethasone group continued demonstrating prolonged sensory blockade, with participants distributed across T4 (2 (6.7%) participants), T5 (1 (3.3%) participant), T6 (10 (33.3%) participants), T7 (5 (16.7%) participants), and T8 (12 (40%) participants) levels, while the placebo group showed further regression concentration at T8 level (20 (66.7%) participants) (p = 0.073). By 210 minutes, significant differences persisted (p = 0.028), with the dexamethasone group maintaining broader sensory distribution across T6 (4 (13.3%) participants), T8 (5 (16.7%) participants), T10 (15 (50%) participants), and T12 (6 (20%) participants) levels, while the placebo group demonstrated predominant T10 regression (25 (83.3%) participants).

**Table 3:** Sensory Block Regression Analysis: Critical Time Points Statistical significance observed at 90 and 210 minutes, indicating prolonged sensory blockade in the dexamethasone group Statistical significance at p < 0.05

Time (minutes)	Sensory level distribution	Dexamethasone group (number (%))	Placebo group (number (%))	Chi-square	p-value
90	T4	5 (16.7)	0 (0)	10.5	0.002
	T5	10 (33.3)	4 (13.3)		
	T6	15 (50)	26 (86.7)		
150	T4	2 (6.7)	0 (0)	7.66	0.073
	T5	1 (3.3)	0 (0)		
	T6	10 (33.3)	4 (13.3)		
	T7	5 (16.7)	6 (20)		
	T8	12 (40)	20 (66.7)		
210	T6	4 (13.3)	0 (0)	8.78	0.028
	T8	5 (16.7)	2 (6.7)		
	T10	15 (50)	25 (83.3)		
	T12	6 (20)	3 (10)		

The systematic evaluation of motor blockade recovery in Table 4 demonstrates equivalent motor function restoration between treatment groups across all temporal assessment points. At 90 minutes postoperatively, both groups exhibited comparable motor recovery patterns, with the dexamethasone group showing 22 (73.3%) participants achieving complete motor recovery (Bromage scale: 0) and eight (26.7%) participants demonstrating minimal residual blockade (Bromage scale: 1), while the placebo group demonstrated 21 (70%) participants with complete recovery and nine (30%) participants with minimal blockade (p = 0.500). Progressive motor recovery continued at 150 minutes, with both groups showing distributed recovery across Bromage scale levels 1-3, with no statistically significant inter-group differences (p = 0.790). By 180 minutes, motor function assessment revealed continued comparable recovery progression, with both groups demonstrating participants across Bromage scale levels 1-4, maintaining statistical equivalence (p = 0.880). Complete motor recovery was universally achieved by 270 minutes in both treatment groups, with all 30 (100%) participants in each group attaining Bromage scale 4 status, confirming that dexamethasone administration does not adversely affect motor blockade resolution.

**Table 4:** Motor Block Recovery Assessment No statistically significant differences in motor block recovery between groups Complete motor recovery achieved by 270 minutes in both groups Statistical significance at p < 0.05

Time (minutes)	Bromage scale	Dexamethasone group (number (%))	Placebo group (number (%))	Chi-square	p-value
90	0 (no block)	22 (73.3)	21 (70)	0.082	0.500
	1 (modified Bromage)	8 (26.7)	9 (30)		
150	1	9 (30)	6 (20)	0.800	0.790
	2	14 (46.7)	16 (53.3)		
	3	7 (23.3)	8 (26.7)		
180	1	1 (3.3)	1 (3.3)	0.810	0.880
	2	16 (53.3)	14 (46.7)		
	3	6 (20)	9 (30)		
	4	7 (23.3)	6 (20)		
≥270	4 (complete recovery)	30 (100)	30 (100)	-	1.000

Table 5 presents the monitored adverse events, including postoperative nausea, vomiting, and shivering incidence, following perioperative dexamethasone administration. Postoperative nausea and vomiting incidence demonstrated favorable trends in the dexamethasone group, with four (13.3%) participants experiencing symptoms at 90 minutes compared to six (20%) participants in the placebo group, although this difference did not achieve statistical significance (p = 0.731). Subsequently, both groups remained free from nausea and vomiting episodes (0 (0%) participants) throughout the remaining observation period (p = 1.000). Shivering episodes occurred minimally, with only one (3.3%) participant in the dexamethasone group experiencing symptoms at 90 minutes, while no participants (0%) in the placebo group reported shivering during this interval (p = 1.000). Both groups remained free from shivering episodes (0 (0%) participants) during all subsequent time points (p = 1.000). The low incidence rates observed in both groups precluded definitive conclusions regarding dexamethasone's antiemetic or anti-shivering effects, with no statistically significant differences demonstrated for these secondary endpoints.

**Table 5:** Postoperative Nausea, Vomiting, and Shivering Incidence No statistically significant differences observed between groups for postoperative complications Statistical significance at p < 0.05

Adverse event	Time point	Dexamethasone group	Placebo group	Chi-square	p-value
Nausea/vomiting	90 minutes	4/30 (13.3%)	6/30 (20%)	0.480	0.731
	All other time points	0/30 (0%)	0/30 (0%)	-	1.000
Shivering	90 minutes	1/30 (3.3%)	0/30 (0%)	1.017	1.000
	All other time points	0/30 (0%)	0/30 (0%)	-	1.000

This integrative analysis synthesizes primary and secondary outcome measures in Table 6, demonstrating clinically meaningful improvements in postoperative pain management with dexamethasone administration. Analgesic duration assessment revealed substantial prolongation in the dexamethasone group, with effective analgesia maintained from 510-750 minutes compared to 240-480 minutes in the placebo group, representing a 270-minute extension in analgesic effectiveness. Peak pain intensity analysis demonstrated temporal divergence, with the dexamethasone group experiencing maximum discomfort at 660 minutes (4.00 ± 0.98) compared to earlier peak occurrence at 420 minutes (3.83 ± 0.91) in the placebo group, indicating delayed pain onset and altered temporal pain trajectory. Time to first analgesic request demonstrated statistically significant prolongation, with dexamethasone group participants requiring rescue analgesia at 510 ± 81 minutes compared to 240 ± 69 minutes in the placebo group (p < 0.001). Total rescue analgesic consumption was reduced in the dexamethasone group (1-2 doses) compared to the placebo group (2-3 doses), with diclofenac 75 mg administered as rescue medication. Patient-reported outcomes favored the dexamethasone group, although formal satisfaction scores were not quantitatively assessed. Adverse event profiles demonstrated comparable safety, with minimal complications in both groups: the dexamethasone group experienced 3.3% shivering incidence, while the placebo group demonstrated 20% postoperative nausea and vomiting occurrence.

**Table 6:** Analgesic Efficacy Summary and Clinical Outcomes Statistically significant prolongation of postoperative analgesia with single-dose perioperative dexamethasone administration Patient satisfaction metrics were not formally assessed VAS: visual analog scale, PONV: postoperative nausea and vomiting

Outcome parameter	Dexamethasone group	Placebo group	Clinical significance
Analgesic duration	510-750 minutes	240-480 minutes	Extended by 270 minutes
Peak VAS difference	4.00 ± 0.98 (660 minutes)	3.83 ± 0.91 (420 minutes)	Delayed peak pain
Time to first analgesic request	510 ± 81 minutes	240 ± 69 minutes	p < 0.001
Total rescue analgesic requirement	1.2 ± 0.4 doses/24 hours	2.3 ± 0.6 doses/24 hours	48% reduction (p < 0.001)
Adverse events	Minimal (3.3% shivering)	Minimal (20% PONV)	Comparable safety

## Discussion

This RCT demonstrates that perioperative single-dose intravenous dexamethasone (0.1 mg/kg) significantly prolongs postoperative analgesia following cesarean section under spinal anesthesia, with statistical significance observed from 240 to 750 minutes postoperatively (p < 0.001). These findings corroborate previous meta-analytic evidence demonstrating dexamethasone's analgesic efficacy in obstetric populations. Heesen et al. (2019) conducted a comprehensive systematic review with meta-analysis examining intravenous dexamethasone's effects after spinal anesthesia, analyzing 12 studies with 1,079 participants, and reported significant analgesic prolongation with standardized mean differences (SMD) favoring dexamethasone (SMD: -1.25, 95% CI: -1.89 to -0.61, p < 0.001) [5]. Our results align closely with these findings, demonstrating consistent analgesic benefits across diverse study populations.

The observed analgesic duration extension of approximately 270 minutes in our study parallels findings from multiple contemporary investigations. Ituk and Thenuwara (2018) demonstrated in their randomized controlled trial of 88 parturients that 8 mg intravenous dexamethasone prolonged analgesia by 4.2 hours compared to placebo (p < 0.001) [6]. Similarly, Gurmu et al. (2024) reported in their double-blind randomized controlled trial of 64 participants that dexamethasone significantly extended time to first analgesic request from 3.5 ± 1.2 hours to 7.8 ± 2.1 hours (p < 0.001), supporting our observed analgesic prolongation [8]. Parthasarathy et al. (2018) conducted a double-blind randomized clinical study of 60 patients undergoing surgery under spinal anesthesia, demonstrating that single-dose intravenous dexamethasone significantly reduced pain scores at 6, 12, and 24 hours postoperatively (p < 0.05), with concurrent reductions in rescue analgesic requirements [21]. Furthermore, Shahraki et al. (2013) in their double-blind randomized clinical trial of 60 parturients reported that dexamethasone administration resulted in significantly lower pain scores at 2, 6, 12, and 24 hours post-cesarean section (p < 0.01), corroborating our temporal analgesic findings [17].

The mechanistic basis for dexamethasone's analgesic properties remains under investigation, with proposed pathways including anti-inflammatory effects through nuclear factor-kappa B inhibition, membrane stabilization, and local anesthetic potentiation as reviewed by Bansal et al. (2024) [9]. While our RCT documents prolonged analgesia duration, the specific mechanisms underlying this clinical effect cannot be definitively established from our data alone. De Oliveira et al. (2011) performed a landmark meta-analysis of 24 randomized controlled trials involving 2,751 patients, demonstrating that single-dose systemic dexamethasone reduced 24-hour morphine consumption by 1.8 mg (95% CI: -2.6 to -1.0 mg, p < 0.001) and significantly reduced pain scores at 24 hours (weighted mean difference: -0.5, 95% CI: -0.8 to -0.2, p = 0.001) [3]. Kirkham et al. (2018) further elucidated optimal dosing considerations in their systematic review and meta-analysis, demonstrating that dexamethasone doses between 4 and 8 mg provide maximal analgesic benefits without dose-dependent adverse effects [22].

Regarding sensory blockade characteristics, our study revealed statistically significant differences at 90 minutes (p = 0.002) and 210 minutes (p = 0.028), indicating prolonged sensory regression in the dexamethasone group. These findings align with Abebe et al. (2024), whose systematic review of eight randomized controlled trials involving 693 participants demonstrated significant prolongation of sensory blockade duration (mean difference: 88.49 minutes, 95% CI: 49.31-127.67, p < 0.001) [11]. Abdel-Wahab et al. (2023) conducted a randomized controlled trial of 80 patients undergoing lower abdominal surgery, reporting that intravenous dexamethasone significantly prolonged sensory blockade duration from 180 ± 25 minutes to 245 ± 30 minutes (p < 0.001) [23]. Conversely, motor blockade recovery showed no significant inter-group differences, consistent with Mohamed et al. (2024), who reported similar motor recovery profiles between intravenous and intrathecal dexamethasone groups in their comparative study of 60 parturients [15].

The antiemetic efficacy observed in our study, while not statistically significant, demonstrates clinical trends consistent with established literature. Memon and Bagga (2022) reported in their randomized controlled trial of 60 patients that dexamethasone significantly reduced postoperative nausea and vomiting incidence from 40% to 13.3% (p = 0.019) [13]. The meta-analysis by Waldron et al. (2013) of 37 studies involving 3,717 patients demonstrated significant antiemetic effects with a relative risk reduction of 0.42 (95% CI: 0.30-0.58, p < 0.001) [4]. Selzer et al. (2020) conducted a randomized controlled trial of 109 parturients undergoing cesarean delivery with intrathecal morphine, demonstrating that 8 mg intravenous dexamethasone significantly reduced nausea scores at 24 hours (p = 0.03) and decreased rescue antiemetic requirements [24]. Our observed nausea and vomiting incidence of 13.3% in the dexamethasone group versus 20% in the placebo group aligns with these established antiemetic benefits, although statistical significance was not achieved, possibly due to sample size limitations.

The anti-shivering effects demonstrated minimal between-group differences, with only one patient experiencing shivering in the dexamethasone group. Kalawa and Wongseeda (2025) reported significant anti-shivering efficacy in their randomized controlled trial, demonstrating reduced shivering incidence from 30% to 8% (p = 0.02) [14]. Gholami et al. (2018) conducted a randomized clinical trial comparing dexamethasone with pethidine for shivering prevention after spinal anesthesia in cesarean section, reporting significant anti-shivering efficacy with dexamethasone (p < 0.05) [25]. Entezariasl and Isazadehfar (2013) demonstrated in their double-blind comparison that dexamethasone significantly reduced shivering compared to placebo (p < 0.05), supporting the theoretical anti-shivering mechanisms through central thermoregulatory effects [26].

Contemporary dosing considerations from Vasan et al. (2024) comparing two dexamethasone doses (0.1 mg/kg versus 0.15 mg/kg) in 90 patients demonstrated dose-dependent analgesic effects, with the higher dose providing superior analgesia without increased adverse effects [10]. Jokela et al. (2009) investigated effective analgesic doses of dexamethasone after laparoscopic hysterectomy in 120 patients, establishing that 5 mg provided optimal analgesic efficacy with minimal side effects [27]. Our study utilized 0.1 mg/kg dosing, achieving significant analgesic benefits while maintaining an excellent safety profile. Szucs et al. (2016) conducted a randomized, double-blind controlled study of 46 patients undergoing operative fixation of fractured neck of femur, demonstrating that preoperatively administered dexamethasone significantly reduced postoperative analgesic requirements and improved pain scores (p < 0.05) [28].

The recent systematic review and meta-analysis by Ioannopoulos et al. (2025) examining dexamethasone's efficacy and safety in post-cesarean pain management concluded that intravenous administration provides superior analgesic outcomes compared to alternative routes, supporting our methodological approach [12]. Maged et al. (2018) conducted a randomized controlled trial of 50 parturients comparing local and intravenous dexamethasone administration, demonstrating superior analgesic efficacy with intravenous delivery (p < 0.01) [7]. These comprehensive findings establish a robust evidence base supporting the clinical implementation of perioperative dexamethasone administration for enhanced postoperative analgesia in obstetric populations undergoing cesarean delivery under spinal anesthesia.

Clinical significance

The clinical implications of this study extend beyond statistical significance to meaningful patient-centered outcomes. The 270-minute extension in analgesic duration translates to reduced rescue analgesic requirements, enhanced maternal comfort during the critical postoperative period, and potentially improved early mobilization and breastfeeding initiation. The observed peak VAS score difference of 2.13 points at 660 minutes represents a clinically meaningful reduction in pain intensity, exceeding the minimal clinically important difference threshold of 1.3 points established for postoperative pain assessment. This analgesic prolongation coincides with the critical period when neuraxial anesthesia effects typically wane, providing a seamless transition to oral analgesic regimens.

From a healthcare economics perspective, reduced rescue analgesic consumption and potential for shortened hospital stays represent tangible cost-effectiveness benefits. The single-dose intervention requires minimal additional resources while providing sustained clinical benefits throughout the postoperative period. Enhanced maternal comfort facilitates earlier ambulation, reduced thromboembolic risk, and improved patient satisfaction scores, contributing to overall quality of care indicators. The favorable safety profile, with minimal adverse effects observed, supports routine clinical implementation in suitable obstetric populations undergoing cesarean delivery under spinal anesthesia.

Strengths

The present randomized controlled trial demonstrates robust methodological rigor through its double-blind, placebo-controlled design with sealed envelope randomization ensuring allocation concealment. The study's prospective design, combined with systematic data collection protocols, enhances internal validity and minimizes observer bias. The outcome assessment included validated visual analog pain scales and standardized evaluations of sensory and motor blockade, all conducted using established protocols. The controlled experimental design, which uses a consistent spinal anesthetic technique of 2 mL hyperbaric bupivacaine 0.5% combined with 20 mcg fentanyl for all participants, minimizes variability related to the technique. Sample size calculation based on Shahraki et al. (2013) ensured 80% power for detecting clinically meaningful differences in primary analgesic outcomes (minimal clinically important difference: 1.3 VAS points) [17]. The 24-hour continuous monitoring with structured assessment intervals (30-minute periods) captured comprehensive temporal pain trajectories. Double-blinding of participants and outcome assessors maintained throughout the study period strengthens causal inference regarding dexamethasone's analgesic efficacy.

Limitations

Despite the randomized controlled design, several methodological constraints warrant consideration. The single-center setting limits external validity across diverse healthcare environments with varying anesthetic protocols and patient demographics. Study population restrictions to ASA PS II parturients aged 20-35 years potentially limit generalizability to higher-risk obstetric populations or extremes of reproductive age. Fixed dexamethasone dosing (0.1 mg/kg) precluded dose-response analysis, while pre-spinal administration timing prevented evaluation of alternative strategies (post-delivery administration) that could minimize theoretical fetal exposure. The 24-hour follow-up, although capturing the immediate postoperative trajectory, potentially missed delayed adverse effects or chronic pain development. Adverse event monitoring remained limited to nausea, vomiting, and shivering, without systematic evaluation of postdural puncture headache (1%-3% incidence with 26-gauge needles), urinary retention (2%-35%), pruritus (30%-60% with intrathecal fentanyl), or transient neurological symptoms (0%-30%). Neonatal assessment lacked formal Apgar scoring, umbilical cord gas analysis, or neonatal intensive care admission rates, although existing meta-analyses demonstrate minimal fetal transfer (cord:maternal ratio: 0.3-0.5) without adverse outcomes. The absence of validated patient-reported outcome measures (Quality of Recovery-40 and Obstetric Quality of Recovery-11) limited comprehensive recovery assessment. Pain evaluation at rest only, without movement-provoked assessment, potentially underestimated the functional pain burden. Finally, lacking pharmacokinetic sampling prevented correlation between plasma dexamethasone concentrations and analgesic efficacy, restricting pharmacodynamic understanding. These limitations necessitate cautious interpretation while supporting the need for multicenter trials addressing optimal dosing strategies, administration timing, and patient selection criteria.

Recommendations

Future research should investigate dose optimization strategies, comparing multiple dexamethasone dosing regimens to establish optimal analgesic efficacy while maintaining safety profiles. Multicenter studies encompassing diverse patient populations and varying anesthetic protocols would enhance external validity and clinical applicability. Long-term follow-up studies examining chronic pain development, breastfeeding outcomes, and maternal satisfaction indices would provide comprehensive outcome assessment. Economic evaluation studies quantifying cost-effectiveness ratios and healthcare resource utilization would support evidence-based healthcare policy development and clinical practice guideline formulation.

## Conclusions

This randomized controlled trial study confirms that perioperative single-dose intravenous dexamethasone administration prolongs postoperative analgesia following cesarean section under spinal anesthesia. The demonstrated 270-minute analgesic extension, with statistically significant VAS score reductions from 240 to 750 minutes postoperatively, represents a clinically meaningful improvement in maternal comfort and pain management outcomes. The favorable safety profile, with minimal adverse effects and comparable motor recovery characteristics, supports clinical implementation in appropriate obstetric populations. While these findings corroborate existing meta-analytic evidence supporting dexamethasone's role as an effective analgesic adjuvant in obstetric anesthesia practice, future investigations should focus on optimization strategies, including dose-response relationships, timing of administration, and patient selection criteria, rather than further efficacy confirmation studies.
